# Auxotrophic interactions: a stabilizing attribute of aquatic microbial communities?

**DOI:** 10.1093/femsec/fiaa115

**Published:** 2020-06-10

**Authors:** Winifred M Johnson, Harriet Alexander, Raven L Bier, Dan R Miller, Mario E Muscarella, Kathleen J Pitz, Heidi Smith

**Affiliations:** MIT/WHOI Joint Program in Oceanography/Applied Ocean Sciences and Engineering, Department of Marine Chemistry & Geochemistry, Woods Hole Oceanographic Institution, 266 Woods Hole Road, Woods Hole, MA 02543, USA; Biology Department, Woods Hole Oceanographic Institution, 266 Woods Hole Road, Woods Hole, MA 02543, USA; Stroud Water Research Center, 970 Spencer Rd., Avondale, PA 19311, USA; PureMagic LTD, Rambam 67, Yad Rambam 9979300, Israel; Department of Plant Biology, University of Illinois, 505 South Goodwin Avenue, Urbana, IL, 61801, USA; Monterey Bay Aquarium Research Institute, 7700 Sandholdt Road, Moss Landing, CA 95039, USA; Center for Biofilm Engineering, Department of Microbiology and Immunology, Montana State University, 366 Barnard Hall, Bozeman, MT 59717, USA

**Keywords:** auxotrophy, microbial community stability, microbial interactions, aquatic

## Abstract

Auxotrophy, or an organism's requirement for an exogenous source of an organic molecule, is widespread throughout species and ecosystems. Auxotrophy can result in obligate interactions between organisms, influencing ecosystem structure and community composition. We explore how auxotrophy-induced interactions between aquatic microorganisms affect microbial community structure and stability. While some studies have documented auxotrophy in aquatic microorganisms, these studies are not widespread, and we therefore do not know the full extent of auxotrophic interactions in aquatic environments. Current theoretical and experimental work suggests that auxotrophy links microbial community members through a complex web of metabolic dependencies. We discuss the proposed ways in which auxotrophy may enhance or undermine the stability of aquatic microbial communities, highlighting areas where our limited understanding of these interactions prevents us from being able to predict the ecological implications of auxotrophy. Finally, we examine an example of auxotrophy in harmful algal blooms to place this often theoretical discussion in a field context where auxotrophy may have implications for the development and robustness of algal bloom communities. We seek to draw attention to the relationship between auxotrophy and community stability in an effort to encourage further field and theoretical work that explores the underlying principles of microbial interactions.

## Teaser

Discussion of the potential impact of auxotrophy, the requirement by an organism for an organic micronutrient, on the stability of aquatic microbial communities in the face of environmental changes.

## INTRODUCTION

**Figure 1.**
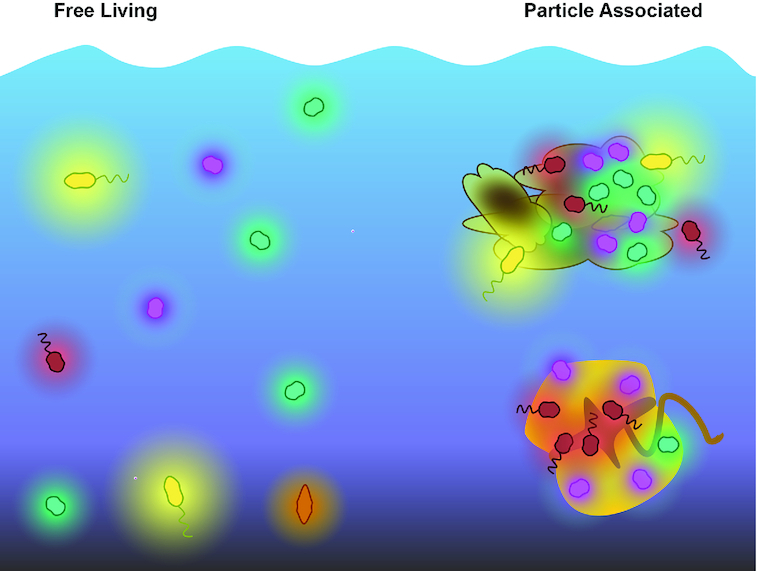
Auxophores (defined in Box 1) diffuse from marine microbes. The proximity of the prototroph to the auxotroph can vary from free-living environments where the auxophore must diffuse through the water before it is located by the auxotroph, to particle-associated communities where the auxotroph and prototroph interact in much closer proximity.

The interactions that link microbial species influence community structure and, consequently, stability (May 1972; Allesina and Tang 2012; Butler and O'Dwyer 2018). Auxotrophy – the requirement for an exogenous source of an organic molecule (Droop 1957) which we designate here as the ‘auxophore’ – results in an obligate interaction between an auxotroph and at least one prototroph (an organism that can synthesize the auxophore). In aquatic ecosystems, auxotrophy has been demonstrated in a variety of microbial species (Table 1; Box 1). These ecosystems contain a diversity of physical environments that determine how microorganisms interact in attached or planktonic phases (Fig. 1). In planktonic aquatic communities, in particular, the distance molecules must travel between microbes makes the occurrence of these obligate interactions surprising, while attached communities are intuitively more conducive to the exchange of small molecules (Fig. 1). However, indications of a prevalence of genome streamlining in aquatic microbes (Giovannoni, Thrash and Temperton 2014), which could result in auxotrophy, suggest that auxotrophic interactions could be an essential component of how these communities function. Despite evidence that auxotrophy may be common (e.g. D'Souza *et al*. 2014; D'Souza *et al*. 2018; Zengler and Zaramela 2018), we still lack the data to determine its true pervasiveness and impact on aquatic microbial communities. Empirically testing the influence of auxotrophy on microbial community structure and stability is challenging, in part due to a lack of complex experimental microbial communities.

**Table 1.** Cases of experimentally confirmed microbial auxotrophy from a variety of aquatic environments.

Compound	Auxotrophic species	Environment	Citation
Cobalamin	Chlorophyta (44/148)*, Rhodophyta (12/13), Cryptophyta (5/6), Dinophyta (24/27), Euglenophyta (13/15), Haptophyta (10/17), Heterokontophyta (47/80)	Open ocean, coastal, freshwater	reviewed Croft *et al*. 2006
Thiamin	Chlorophyta (19/148)*, Cryptophyta (5/6), Dinophyta (7/27), Euglenophyta (11/15), Haptophyta (14/17), Heterokontophyta (11/80)	Open ocean, coastal, freshwater	reviewed Croft *et al*. 2006
Biotin	Cryptophyta (1/6)*, Dinophyta (7/27), Euglenophyta (1/15), Heterokontophyta (5/80)	Open ocean, coastal, freshwater	reviewed Croft *et al*. 2006
4-amino-5-hydroxymethyl-2- methylpyrimidine	SAR11	Open ocean	Carini *et al*. 2014
Glycine, serine	SAR11	Open ocean	Tripp *et al*. 2009
Isoleucine, valine	*Pseudomonas syringae*	Antarctic freshwater	Sahu and Ray 2008

* Refers to the number of auxotrophic species out of the total number of species surveyed by Croft *et al*. 2006.

**Box 1: Initial identification of auxotrophy in the marine environment**

The word auxotrophy is derived from the Greek, meaning ‘to increase nourishment’. In biology it refers to the requirement of an exogenous source of an organic molecule. In the beginning of the 20th century, considerable efforts were made to develop methods for the cultivation of pure diatom cultures, a demand driven by the desire to supply fisheries with a stable food source. It was discovered that diatoms failed to thrive on artificial sea water, with E. J. Allen of Plymouth Marine Laboratory noting: ‘Stated in general terms the most interesting result so far obtained is that in the artificial sea-water tried… little or no growth of diatoms… takes place, but if to this artificial sea-water as little as 1% of natural sea-water is added vigorous and large culture are obtained…’ (Allen 1914). Moreover, different natural seawater sources were found to differentially impact the growth of diatom cultures. The phenomenon was surprising, as it was previously thought that diatoms were self-sufficient, and was attributed to the newly discovered vitamins (Funk 1912). This discovery in diatoms was the first of many observations that found that a wide diversity of organisms required additive compounds in order to successfully thrive and grow.

**Figure 2.**
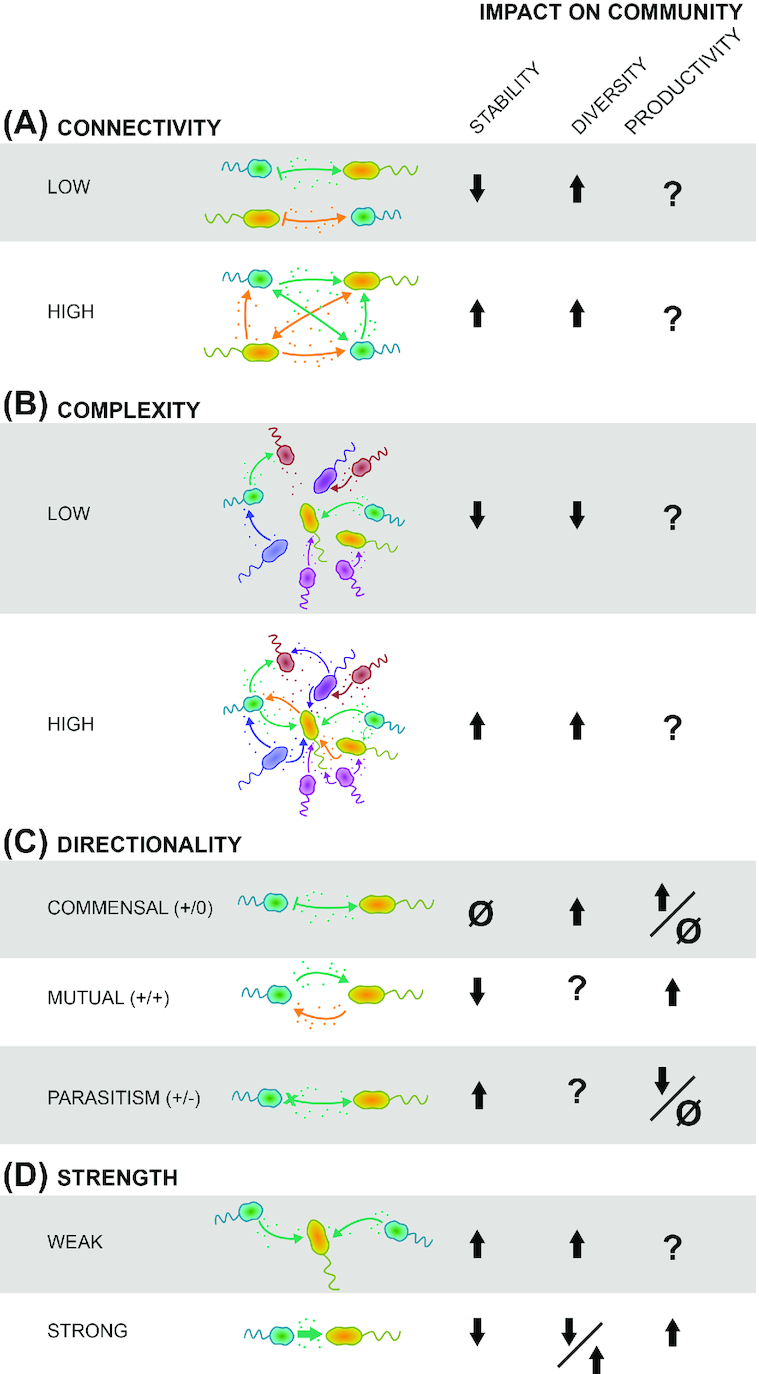
The potential impacts (↑, increase; ↓, decrease; Ø, no change) of auxotrophy on community stability, diversity and productivity across a variety of community states and individual interactions types. See references in text.

While the strength and complexity of network interactions are known to play a role in the resistance and resilience of ecological communities, auxotrophy specifically has not been considered in this context (reviewed by Shade *et al*. 2012). Given that an auxotrophic requirement must be fulfilled for the organism to survive, this suggests an extreme vulnerability in the organism especially in less spatially structured aquatic environments. However, auxotrophy is a successful evolutionary strategy. This paper explores how auxotrophy might contribute to the stabilization of aquatic microbial networks over the long-term and highlights areas for future research. A number of factors must be considered to understand the relationship between auxotrophy and stability, including (i) the connectedness of the auxotroph to other community members that are the sources of auxophores, (ii) the complexity of the microbial community (i.e. the number and type of interactions), (iii) the extent to which auxotrophy results in a positive or negative feedback loop through cooperative or competitive interactions and (iv) the strength of the relationship between the auxotroph and prototroph (i.e. is there only one prototroph producing the auxophore or are there multiple sources) (Fig. 2). We define and discuss the extent to which these factors are applicable for understanding auxotrophy across aquatic microenvironments, while also recognizing that these factors can be influenced by the physical association of a microbe (whether associated with a particle, biofilm or non-associated free-living). Based on the existing research, we propose that auxotrophy will stabilize microbial communities if it results in a high degree of network complexity, redundant prototrophy and competition keeping the population abundance in check. We believe that these stabilizing characteristics are likely present in many aquatic microbial communities.

## AUXOTROPHY-CONFERRED EVOLUTIONARY FITNESS

Auxotrophic organisms depend on other community members for essential metabolites, thereby creating interspecies interactions that can alter the diversity and structure of microbial food webs (Seth and Taga 2014). Although auxotrophy increases an organism's dependence on the rest of the community, it can be evolutionarily favorable and, thus, maintained in a population under certain circumstances. This has been reviewed extensively elsewhere (D'Souza *et al*. 2018) and therefore, will be discussed only briefly here. According to the Black Queen Hypothesis (BQH: Morris, Lenski and Zinser 2012), species can gain a fitness advantage through genome streamlining (Giovannoni, Thrash and Temperton 2014). This reduces the nutrient requirements associated with the maintenance of more genetic material and limits energetically costly metabolic activities. Organisms living in an environment where a particular organic compound is freely available may be more likely to accumulate mutations, including loss-of-function mutations, in genes involved in the biosynthesis of the available compound (D'Souza and Kost 2016). These mutant subpopulations would have higher fitness than wild type subpopulations that still require resources to produce the compound (van de Guchte *et al*. 2006; Lynch and Marinov 2015; D'Souza and Kost 2016). These loss-of-function mutations become increasingly probable under environmental conditions that support or even induce mutagenesis, as observed under starvation, high UV irradiation, and other stressors (Adelberg, Mandel and Chen 1965; Foster 1993). The evolutionary advantage conferred by loss-of-function mutations has been demonstrated in a laboratory setting in which amino acid auxotrophs of *Escherichia coli* emerged in less than 2000 generations of growth in the presence of amino acids (D'Souza and Kost 2016) indicating strong selection for these types of mutations under the correct conditions. Although we might expect these conditions to occur more frequently in populations of aquatic microbes that are physically associated with each other, there is strong evidence that auxotrophy also develops in free-living aquatic populations (see section on *Auxotrophy in aquatic environments*).

## AUXOTROPHY AND COMMUNITY STABILITY

The stability of an ecological community (Box 2) A is qualitatively defined as the community's ability to return to or resemble its pre-existing state following a disturbance (Loreau 2010). It has been broken down into a series of properties that can be quantified or parameterized and include resilience, resistance, robustness, variability, and persistence (see Loreau, Naeem and Inchausti 2002 for extended list; see Box 3 for definitions). These properties can be used to describe community variability through time, and specifically the response of a community to a disturbance such as a pulse (short-term, e.g. nutrient inputs due to storm events) or press (long-term, e.g. climate change) perturbation (Bender, Case and Gilpin 1984). Stability can be considered a spectrum where a community may be relatively resilient and can thus recover from a mild disturbance but perhaps not from a more severe or prolonged disturbance (Shade *et al*. 2012). A meta-analysis that investigated the stability of microbial communities in 247 studies found that across a variety of ecosystems (ranging from terrestrial to marine to gut) microbial communities are sensitive to both pulse and press perturbations with 82% of communities responding to disturbance; however, relatively few of the studies assessed community resilience and recovery (Shade *et al*. 2012). A variety of features might contribute to resilience in the face of changes, such as phenotypic plasticity and compositional turnover of the community (Shade *et al*. 2012). While the potential connection between auxotrophy and stability has not been extensively examined, we discuss auxotrophy's potential impact on microbial communities in the context of the larger debate surrounding how complexity, interactions (strength and direction), and the degree of community connectedness affect stability. Furthermore, we expect that these factors cannot be considered in isolation from one another – they can work together or in opposition to change a community's overall stability, and one factor may influence another: for example, the connectedness of a community could affect the degree to which certain interactions affect its stability. In the following sections we discuss how auxotrophy's effects on community connectivity, complexity, interaction directionality, and strength may affect aquatic microbial community stability.

**Box 2: Defining microbial communities**

When considering the impact of auxotrophy on microbial community structure one must first define the time and scale of microbial community interactions. Communities are defined by the composition, diversity, and abundance of species inhabiting the same space and time (Vellend 2010). The scale at which microbes interact with their environment is far different than that of their macroscopic analogs (Konopka 2009), with microbes interacting with and influencing a small space immediately surrounding the microbe, often termed a microenvironment (Stocker 2012). Notably, even within the microbial world the range of relevant spatial and temporal scales vary by system (e.g. the relevant time and spatial scales that best encapsulate the microbial community of a gut are likely to be much shorter and smaller than that of deep sea sediments). In spite of their narrow sphere of individual influence, microbial communities can drastically alter their macro-environment, for example microbial communities are known to form chemical gradients in poorly mixed or stratified environments (e.g. mudflats) through the consumption and production of substrates (Nealson 1997). Therefore, how auxotrophic interactions shape microbial community structure may have a direct influence on the community's environmental chemical composition on both macro and micro scales.

**Box 3. Definitions**

Auxophore: The compound (vitamin, sugar, etc.) that is required for growth by an auxotroph; the compound that is exchanged in an auxotrophic interaction. (Term originating here with the current article.)

Auxotrophy: The inability of an organism to synthesize a particular organic compound required for its growth (IUPAC).

Black Queen Hypothesis: The hypothesis that species gain an advantage by undergoing evolutionary reduction of genome size, thereby reducing nutrient requirements associated with the maintenance of more genetic material and limiting energetically costly metabolic activities (Morris, Lenski and Zinser 2012).

Commensal ism: A symbiotic interaction between organisms in which one benefits and the other is neither positively nor negatively influenced. In the context of this paper, one auxotroph receives an essential compound synthesized by a prototroph without a positive or negative consequence to the prototroph.

Community stability: The ability of a system to return to its original state after perturbation.

Cross-feeding: A molecule produced selfishly by one organism becomes available extracellularly and is consumed by another organism. This interaction can be a commensalism if unidirectional or a mutualism if bidirectional via another molecule or other benefit.

Higher-order interactions: Interactions between more than two species.

Mutualism: A symbiotic interaction between organisms in which both benefit. In the context of this paper, at least two auxotrophs complement each other by exchanging an essential compound which one has synthesized and the other organism cannot.

Negative feedback: A type of response where an increase in one subject causes a decrease in one responder (inhibits).

Parasitism /Exploitative: A type of interaction between organisms in which one benefits but the other is negatively impacted.

Persistence: “A measure of the ability of a system to maintain itself through time. Generally used for non-equilibrium or unstable systems before extinction occurs" (Loreau, Naeem and Inchausti 2002).

Positive feedback: A type of response where an increase in one subject causes an increase in the responder (amplifies).

Prototrophy: The capability to synthesize an organic compound required for growth.

Resilience: “A measure of the speed at which a system returns to its original state after a perturbation (Webster *et al*. 1975) Generally used for an equilibrium state, though it could also be applied to systems that return to non-equilibrium trajectories" (Loreau, Naeem and Inchausti 2002).

Resistance: “A measure of the ability of a system to maintain its original state in the face of an external disruptive force (Harrison 1979). Generally used for an equilibrium state" (Loreau, Naeem and Inchausti 2002).

Robustness: “A measure of the amount of perturbation that a system can tolerate before switching to another state. Closely related to the concept of ecological resilience *sensu* Holling (1973). Can be applied to both equilibrium and non-equilibrium states" (Loreau, Naeem and Inchausti 2002).

Variability: “A measure of the magnitude of temporal changes in a system property. A phenomenological measure which does not make any assumption about the existence of an equilibrium or other asymptotic trajectories" (Loreau, Naeem and Inchausti 2002).

Vitamin: An organic micronutrient which is required for growth. Auxotrophs commonly lack the ability to synthesize this type of compound.

### Community connectivity and complexity

The relationship between community complexity (i.e. the number and types of interactions) and stability is still a matter of debate in ecology (McCann 2000). On the one hand, it is proposed that a high number of weak interactions can stabilize a community, shielding it from boom-bust dynamics caused by strong consumer-resource interactions (Fig. 2A, Fig. 2D; Odum 1953; Polis and Strong 1996). Ecological theory predicts that complex systems are stable as long as negative feedbacks prevent large fluctuations (boom-bust cycles) resulting from positive feedback loops (Fig. 2B; Allesina and Pascual 2008; Coyte, Schluter and Foster 2015). Furthermore, communities with high diversity are considered more stable and resistant to explosive rapid changes because individual species would respond differently to environmental perturbations (reviewed in McCann 2000). On the other hand, mathematical models of communities using random interaction matrices contradict these findings, showing instead that increased species diversity can destabilize communities and higher connectedness can lead to instability (May 1972). Since these models appear to directly contradict the abundance of natural diverse communities found across ecosystems, researchers have been exploring how the type and strength of interactions can permit a large number of species to stably coexist (Allesina and Tang 2012, Butler and O'Dwyer 2018). However, as much of this research is based on models, it should also be recalled that random matrices may not accurately represent interactions in natural microbial communities and do not provide or suggest any biological mechanism.

Complex communities often rely on relatively few keystone species (existing in low or high abundances) that provide essential functions to the majority of other community members. In these complex communities, species can become organized around a few highly connected hub species (Paine 1966; Paine 1969; Cottee-Jones and Whittaker 2012; Morris, Lenski and Zinser 2012, Berry and Widder 2014). We assert that, in addition to classical keystone species such as apex predators, auxotrophic interactions could also generate keystone species relationships when an organism is the sole producer of an essential compound. For example, while it has not been documented as directly supporting auxotrophy, the marine diazotroph *Trichodesmium* can be a vital source of nitrogen and carbon to a complex ‘mat’ community (Hewson *et al*. 2009). However, if the sole producer is lost, then the entire community will drastically respond or even crash. Fortunately, microbial systems commonly have some degree of redundancy in the production of a compound and in the genetic capability to produce these compounds (Louca *et al*. 2018). In addition, the production of these molecules in some cases might not require large additional energy inputs (Pacheco, Moel and Segre 2019) or be static as horizontal gene transfer (HGT) could enable transfer between species (McDaniel *et al*. 2010). Therefore, the degree of functional and genetic redundancy present for certain compounds could mitigate the effects on the community from the loss of a sole producer species (prototroph).

Indications of how auxotrophy may shape the interactions within an aquatic microbial community can be found in network mapping of co-occurring microbial taxa. Prototroph and auxotroph taxa have been observed to co-vary temporally and spatially in microbial community datasets (Aylward *et al*. 2015; Milici *et al*. 2016). Within bacterial networks, some nodes that show a high number of connections to other taxa are taxa with more streamlined genomes, which is a condition that can result in auxotrophy (Peura *et al*. 2015). Peura *et al*. suggest that taxa that are located at convergence points within a network, may confer resistance to disturbance (i.e. stability) when a taxon is removed from the network (2015). It is worth further study to determine if auxotrophic bacteria that maintain a high degree of connectedness within aquatic bacterial communities serve as community stabilizers.

### Interaction directionality

The directionality of an auxotrophic interaction will also play a role in whether the community is stabilized. The possibilities include the classic ecological interaction types: (i) Commensalism, in which the auxotroph is gaining fitness by losing the production of costly metabolites while the provider is not gaining an advantage nor being harmed from the interaction. (ii) Parasitism, where the provider is losing fitness as a result of the interaction. (iii) Mutualism, in which both partners are gaining fitness from the interaction (Fig. 2C). In addition, species may compete or cooperate in obtaining auxophores. A fundamental concept in community ecology is that negative feedbacks (interactions where an organism has a negative impact on another organism; e.g. through competition) prevent unchecked growth through negative frequency dependent selection while positive feedbacks (e.g. through cooperation) accelerate changes and can lead to boom-bust dynamics that are detrimental to community stability (Ross-Gillespie *et al*. 2007; Allesina and Tang 2012; Mougi and Kondoh 2012; Coyte, Schluter and Foster 2015). In short, a key component in determining whether an interaction, like auxotrophy, will stabilize or destabilize a community is if it will reinforce negative or positive feedback loops in a community by dampening or amplifying changes in organism abundance (Holling 1973). While the only interaction type guaranteed to stabilize populations is intra-specific competition, other interaction types can also lead to stability (Chesson 1990; McPeek 2019). As the proportion of competitive interactions increases within a community (assuming no competitive exclusion), it becomes more stable as there is less risk of positive feedback loops (Coyte, Schluter and Foster 2015). As such, the stability of the community results from the additive (or net) effects of all interactions and feedbacks and therefore depends on the proportion of positive and negative interactions and their relative strengths. In the context of auxotrophy, competitive interactions could occur when multiple auxotrophic species compete for an auxophore, similar to an indirect competitive interaction (Holt 1984). Likewise, higher-order interactions (i.e. interactions between more than two species) and secondary resource competition may provide additional negative feedback on the cooperating species (Grilli *et al*. 2017; Muscarella and O'Dwyer 2019). Therefore, while cooperative interactions may increase the productivity of a community by increasing the sharing of resources and division of labor, these gains may come at a cost to community stability unless other forces prevent positive feedback loops (Coyte, Schluter and Foster 2015).

Depending on the extent to which auxotrophy-based interactions are characterized by mutualism (which benefits both auxotroph and prototroph), commensalism (which only benefits the auxotroph), or parasitism (that harms the donor) we would predict that auxotrophy could affect community stability positively or negatively. In the case of mutualism (i.e. +/+ cooperative interaction) auxotrophy would negatively affect the stability of the microbial community by creating a positive feedback loop (Coyte, Schluter and Foster 2015). In the case of commensalism (+/0) only one partner benefits, therefore we predict it will be less detrimental to the stability of the community than mutualistic interactions, although this is a debated topic (Mougi 2016). Exploitative interactions (+/−), where the producer is harmed by the auxotroph, can be considered to affect community stability similarly to competitive interactions by increasing stability (Coyte, Schluter and Foster 2015).

Mutualism is a frequent form of interaction emerging from auxotrophy (Schink 2002; Morris *et al*. 2013). This interaction is strong and persistent due to the interdependence between interaction partners which prevents either of them from overtaking the other, and because it provides both partners with the advantage of losing a biosynthetic pathway (Morris, Lenski and Zinser 2012; Zelezniak *et al*. 2015). Using an engineered synthetic consortium composed of two cross-feeding *Saccharomyces cerevisiae* strains, it was shown that such co-cultures reach a strictly defined (and narrow) range of cell densities after a few days regardless of the initial starting ratios of the two strains. Moreover, this two-strain system is stable even when subjected to massive dilution events that mimic perturbations occurring in natural populations (Shou, Ram and Vilar 2007). Similarly, *E. coli* cultures which cross-feed specific amino acids have been shown to have a significant fitness advantage compared with metabolically-autonomous prototype cells (Pande *et al*. 2014). Mathematical models are also used to describe observed auxotrophic mutualisms which share, for example, vitamin B_12_ and algal photosynthate (Grant *et al*. 2014). Due to the bi-directional interdependence, negative frequency-dependent selection within co-cultures prevented the dominance of ‘non-cooperating competitors’, which would consume the provided amino acids but not donate any (Pande *et al*. 2014). Importantly, in natural populations, this could result in reduced diversity since the population will be dominated by cooperating cross-feeders, and might lead to general destabilization because of abundant positive feedback interactions.

Commensal interactions include donor species that exhibit leaky and/or active production of essential compounds, such as in cross-feeding and therefore allow the occupation of new niches by auxotrophic species (Seth and Taga 2014; Morris 2015). Metabolic modeling suggests that there are an array of metabolites that can be released without a fitness cost to the microbe producing them (Pacheco, Moel and Segre 2019). Commensal interactions between auxotrophs and prototrophs may be an example of a mutualism evolving. Over time, unidirectional cross-feeding from a prototroph's metabolic leakiness (e.g. Rosenzweig *et al*.’s (1994) study of preferential acetate-consumers co-existing with *E. coli* which leak acetate as a glucose metabolism by-product) may give rise to a new metabolic inter-dependency and finally to bidirectional cooperative cross-feeding (reviewed in D'Souza *et al*. 2018). Again, due to the lack of an explicit negative feedback, commensal interactions may also lead to community destabilization.

Auxotrophic organisms could also be parasitic (+/−; Fig. 2C). While this will not inevitably reduce the number of species, it will add a new burden on the prototroph by (i) forcing it to expend more energy producing the required nutrient or (ii) harming it with a toxin to force the release of required molecules (e.g. Seyedsayamdost *et al*. 2011). Parasitisms specific to algae–bacteria interactions have been studied for some time (Ramanan *et al*. 2016), particularly in the context of control factors for algae blooms or in the biotechnology sector for microalgae production. One such example is that of an algae-associated species of the fungus *Exophiala* which when co-cultured in a carbon-free medium with the alga *Chlorella vulgaris*, produced algicides killing *C. vulgaris* (Cho *et al*. 2015). Previously mutualistic interactions can also switch to parasitism following a shift in environmental factors (reviewed by Ramanan *et al*. 2016). This change of directionality has also been documented based on growth phases for the interaction between the marine alga *Emiliania huxleyi* and bacterium *Phaeobacter gallaeciensis*. Here, a mutualistic relationship where the bacterium receives algal carbon and synthesizes growth-promoting molecules and vitamins, becomes pathogenic as the alga ages and the bacterium releases toxins to lyse algal cells (Seyedsayamdost *et al*. 2011). The dinoflagellate *Prorocentrum minimum* and alphaproteobacterium *Dinoroseobacter shibae* are another pair that exhibit a similar relationship in co-cultures (Wang *et al*. 2014). Due to the balancing symmetry of interaction types and feedbacks, the parasitic interaction should stabilize the community.

In addition to considering specific interaction types, it is also important to take into account how community stability may be affected by the diversity of interactions in community and multi-species interactions. Studies performed on pairwise and higher-order combinations of cross-feeding amino acid auxotrophs helped to determine the effects of these interactions on complex synthetic communities (Mee *et al*. 2014). These studies showed that auxotrophic cross-feeding has a positive effect on growth potential (the number of individuals the community is able to sustain) and that complex communities consisting of higher-order auxotrophic interactions have an additional, yet to be explained, effect on the growth potential for all members of the community (Mee *et al*. 2014). Regardless, secondary interactions – including higher-order interactions and secondary resource competition – may be stabilizing even when the auxotrophic interaction alone is predicted to be destabilizing.

### Interaction Strength

In addition to the type or directionality of an interaction, interaction strength also plays a large role in determining how an interaction affects community stability. Interaction strength refers to the size of the effect of one organism on another. In general, weaker interactions lead to greater community stability as they decrease the power of positive feedback loops by reducing the impact of any single organism's change in abundance (Fig. 2D; May 1972; Allesina and Tang 2012; Coyte, Schluter and Foster 2015; Jacquet *et al*. 2016). Therefore, the central question of how auxotrophy influences community stability depends not only on interaction type, but also on whether auxotrophy causes stronger or weaker species interactions in the overall community. Since auxotrophic interactions inherently involve compounds essential for survival, intuitively one would expect a strong interaction between an auxotroph and its prototroph producer. For example, one can imagine that if an auxotroph depends on a metabolite from a single producer (Fig. 2A), then the interaction between the auxotroph and its provider will be essential for the auxotroph's growth and survival. Such interactions hamper community stability, especially if these vital functions are produced by a single keystone species or a limited number of community members. If the abundance of the producer(s) changes drastically and the auxotroph is only limited by this interspecies interaction, then the auxotroph will also change in abundance and overall stability will be impaired. However, auxotrophic interaction networks may also act to weaken these associations. For example, communities have been observed to potentially contain multiple auxotrophies for a variety of auxophores that have multiple sources, creating complex ‘interactomes’ where an auxotroph for one compound may be the prototroph in another auxotrophic relationship (Embree *et al*. 2015; Garcia *et al*. 2015; Hubalek *et al*. 2017). In terms of a single compound, indirect exchanges (i.e. through the production of ‘public goods’) with multiple producers could also create plastic interactions where the identity of the prototroph is not essential, as was hypothesized in Garcia *et al*. (2018). In both scenarios these complex networks have the effect of increasing the number of participants in an auxotrophic interaction and decreasing the strength of any one paired interaction, leading to increased community stability. This conclusion is supported by the finding that higher-order interactions can stabilize the community (Grilli *et al*. 2017; Levine *et al*. 2017) and might be why we see such high diversity in natural communities despite modeled predictions (Fig. 2A).

Many studies of microbial interactions occur in environments where organisms’ interaction strength has a spatial component such as in particle-associated and biofilm communities where microbial cells can be segregated or have greater physical proximity (Coyte, Schluter and Foster 2015). In aquatic environments, planktonic communities also need to be considered and may have the challenge of experiencing greater spatial segregation from interaction partners through dilution or advection, but conversely could have the opportunity to interact with a more diverse array of taxa depending on the concentration of organisms (Stocker 2015; Hein *et al*. 2016; Stump, Johnson and Klausmeier 2018). Either consequence could weaken auxotrophic interaction strength in aquatic environments. Furthermore, horizontal gene transfer and the decoupling of function and species identity would contribute to the weakening of positive feedback loops. Therefore, the effect of auxotrophy within planktonic aquatic communities may be a special case relative to communities in other environments.

While direct evidence is still limited, the combined results of existing theoretical, modeling, experimental, and environmental studies suggest that auxotrophy could play an important role in stabilizing aquatic microbial communities. A study examining microbial genetic data from soil, water, and human gut samples showed that despite a high degree of metabolic competition between species in most communities, sub-populations of taxa that are highly cooperative tended to co-occur across different microbial communities (Zelezniak *et al*. 2015). Based on the evidence presented here, we predict that a complex network with a large number of weak interactions that limits unchecked growth of individual populations through competition will be most stable. Analysis of the co-occurrence of aquatic planktonic bacteria suggests that potential auxotrophs may act as nodes in bacterial networks, relying on a large number of other taxa for provision of auxophores (Peura *et al*. 2015). This scenario fits our expectations of what would be a stabilizing type of interaction (*i.e*. a large number of weak interactions and high community complexity). This network could consist primarily of commensal interactions through the sharing of a pool of metabolites released by community members that do not pay a fitness cost by doing so (Pacheco, Moel and Segre 2019).

## AUXOTROPHY IN AQUATIC ENVIRONMENTS

Several cases of natural microbial auxotrophy have been extensively characterized in aquatic environments (Croft *et al*. 2005; Giovannoni *et al*. 2005; Croft, Warren and Smith 2006; Bertrand *et al*. 2007; Tripp *et al*. 2008; Tripp *et al*. 2009; Koch *et al*. 2011; Bertrand and Allen 2012; Carini *et al*. 2014; Durham *et al*. 2015; Paerl *et al*. 2017; Paerl *et al*. 2018), yet fundamental questions remain such as: How prevalent is auxotrophy in aquatic ecosystems? What is the full canon of auxophores and what are the benefits of auxotrophy for these molecules? How do these requirements shape aquatic microbial community composition and interactions?

The most commonly documented auxophores in the experimental literature across all microbial systems are amino acids and B-vitamins, although this may be biased by a selective focus on these biosynthetic pathways (Table 1). Metabolic modeling of Gram-negative host-associated bacteria has shown that amino acid auxotrophy is often specific to a single metabolite, while nucleotides can be synthesized through a variety of routes resulting in nonspecific auxotrophy (i.e. any of a selection of metabolites can be used for growth) (Seif *et al*. 2020), perhaps explaining why amino acid auxotrophy has often been documented. In nature, auxotrophic interactions may be prevalent: an analysis of 979 metabolic networks predicted that 76% of bacterial genomes were auxotrophic for at least one metabolite. In this analysis the most common auxophores were biotin, phenylalanine, and asparagine (D'Souza *et al*. 2014). This also holds true in aquatic ecosystems where auxotrophies for B vitamins, particularly cobalamin, biotin, and thiamin, have been documented in a variety of phytoplankton species (reviewed by Croft, Warren and Smith 2006). While no eukaryotes can synthesize cobalamin (Warren *et al*. 2002), many have developed alternatives such as a cobalamin-independent methionine synthase that negates the requirement for the cofactor altogether. However, while some marine algae only have the cobalamin-dependent version of the enzyme making them cobalamin auxotrophs (Croft *et al*. 2005; Bertrand *et al*. 2012), none have been identified that exclusively use the cobalamin-independent enzyme (Bertrand *et al*. 2013). This indicates that the cobalamin-dependent version of the enzyme must be favorable in some way and that cobalamin can be obtained exogenously (Bertrand *et al*. 2013). Analysis of the Integrated Microbial Genome (IMG) database suggests that only a few microbes across all the domains of life can synthesize all 20 proteinogenic amino acids (Mee and Wang 2012) indicating that amino acids are significant in these obligate molecular exchanges. A metagenomic study of a freshwater model community proposed that the ubiquitous Actinobacteria are auxotrophic not only for a range of amino acids but also for additional B vitamins such as riboflavin, niacin, pantothenate and folate (Garcia *et al*. 2015), as has also been subsequently shown in the full genomes (Kang *et al*. 2017; Neuenschwander *et al*. 2018). A survey of metagenomes in natural aquatic environments also identified that bacterioplankton are primarily B_1_ auxotrophs (Paerl *et al*. 2018). However, genomic data cannot conclusively indicate auxotrophy due to the likelihood of unknown biosynthetic pathways. For example, an examination of 10 heterotrophic bacteria known to grow without exogenous amino acids but with incomplete amino acid pathways, according to genome annotations, found alternative enzymes indicating that these organisms were not in fact amino acid auxotrophs (Price *et al*. 2018). Experimental evidence has demonstrated that the heterotrophic marine bacterium *Candidatus Pelagibacter ubique* is auxotrophic for glycine but that this requirement can also be met with serine (Tripp *et al*. 2009). Thus, conclusions drawn exclusively from genomic data must be treated cautiously.

While most research has focused on auxotrophy for the final product of a biosynthetic pathway, such as an amino acid or vitamin, there is growing evidence that auxotrophy for specific precursors of biomolecules may be a strategy that some microbes adopt. An example of this is auxotrophy for corrinoids which are precursors for vitamin B_12_ and other biomolecules (reviewed by Seth and Taga 2014). A non-aquatic bacterium, *Listeria innocua*, requires the molecule alpha-ribazole to complete the synthesis of vitamin B_12_ (Gray and Escalante-Semerena 2010). While auxotrophy for this molecule has not been demonstrated in the ocean, alpha-ribazole can be produced by a marine bacterium making such an interaction possible (Johnson, Kido Soule and Kujawinski 2016). Another example of precursor auxotrophy is found in *Candidatus Pelagibacter ubique*, which requires the pyrimidine precursor to thiamin to complete biosynthesis (Carini *et al*. 2014). These precursors widen the array of possible molecules that microbes might need to satisfy their nutritional requirements.

Culturing is required to confirm auxotrophy in an organism. Yet, it remains difficult to isolate and culture most aquatic microbes axenically, potentially because of unknown auxotrophic requirements (Steen *et al*. 2019). Organisms adapted to oligotrophic environments with streamlined genomes and more metabolic dependencies are especially difficult to isolate, particularly under standard culturing procedures (Giovannoni and Stingl 2007; Pande and Kost 2017). Metagenomic surveys have suggested that streamlined genomes are prevalent in the oligotrophic open ocean (Button and Robertson 2001; Swan *et al*. 2013; Giovannoni, Thrash and Temperton 2014; Eiler *et al*. 2016), and may be indicative of instances of auxotrophy. Sequencing and untargeted metagenomics do provide tools to identify putative auxotrophic community members. Such an approach was used to assemble the complete genome of an uncultured organism in the SAR86 clade, which suggested auxotrophy for B-vitamins as well as for the amino acids methionine, histidine, and arginine (Dupont *et al*. 2012). Some groups have used metagenomic data from mixed cultures or model communities to explore auxotrophic relationships (Garcia 2016). For example, researchers used mixed cultures derived from freshwater lakes to describe the networks of the most abundant bacteria and their proposed metabolite exchanges, consisting mostly of vitamins, amino acids, and reduced sulfur molecules (Garcia *et al*. 2015; 2018). These types of studies provide new ways to advance our understanding of the role of obligate cross-feeding interactions in the environment and identification of the molecules that are the currency of these exchanges. But beyond identifying these interactions is the challenge of understanding their impact on aquatic microbial communities in terms of their diversity, stability and role in biogeochemical cycles.

## AVAILABILITY OF AUXOPHORES IN AQUATIC ENVIRONMENTS

Well-described microbial auxophores, such as B vitamins and amino acids, have been measured in aquatic environments, though not extensively (see Table 2 for list of aquatic concentrations of known auxophores) (Sañudo-Wilhelmy *et al*. 2012; Heal *et al*. 2014; Kido Soule *et al*. 2015). This undersampling is due in part to the wide diversity of auxophores as well as their frequently low concentrations in the environment. Vitamins and amino acids, in particular, have been quantified in marine and freshwater environments using a variety of methods (Sañudo-Wilhelmy *et al*. 2012; Heal *et al*. 2014; Kido Soule *et al*. 2015). In the ocean B-vitamins show a nutrient-like profile, in which they are highly depleted in the surface but increase with depth. Dissolved concentrations of up to 30 pM have been measured for vitamin B_2_ and B_12_. Other B-vitamins, including B_1_, B_6_ and B_7_, reach concentrations of ∼500 pM (Sañudo-Wilhelmy *et al*. 2012). In lakes vitamin B_12_ concentrations of ∼3–10 pM have been measured in the surface (Daisley 1969). Moreover, the uptake kinetics for B-vitamins have been measured in natural communities. In eutrophic systems average uptake rates for B_12_ and B_1_ were 3.07 ± 0.57 and 14.4 ± 0.79 pmol L^−1^ day^−1^ across a seasonal time series, respectively (Koch *et al*. 2012). Interestingly, while primary production was concentrated in the larger size fraction (>2 µm), nearly 70% of the uptake of both B-vitamins was concentrated in the pico-plankton (0.2–2 µm) (Koch *et al*. 2012). Dissolved free amino acids are more abundant than vitamins in aquatic environments, reaching low nanomolar concentrations in the surface ocean (Lee and Bada 1975; reviewed by Nagata 2008). In addition, larger peptides and proteins can be hydrolyzed to become bioaccessible, providing a larger pool of amino acids, 20–60 nM for each amino acid (Lee and Bada 1975). Leucine uptake rates have been shown to be species specific and concentration dependent, making uptake rates dependent on both the composition of the community as well as the abundance of the amino acid (Alonso and Pernthaler 2006). In a study in a coastal pelagic environment community uptake rates of radiolabeled leucine ranged from ∼5–100 pmoles L^−1^ h^−1^ (Alonso and Pernthaler 2006). Of course, in contrast to B vitamins, amino acid uptake supports cellular respiration and nitrogen requirements in addition to fulfilling any specific requirement for an individual amino acid. Nevertheless, relatively stable supplies of these molecules could make auxotrophy a beneficial strategy for aquatic microorganisms.

**Table 2.** Existing data on the dissolved concentrations of potential auxophores in aquatic environments.

Compound	Dissolved (pM)	Reference
Biotin	10–200*	Sañudo-Wilhelmy *et al*. 2012
Isoleucine	790–8000^§^	Mopper and Lindroth 1982
Phenylalanine	400–4000^§^	Mopper and Lindroth 1982
Pyridoxine	50–450*	Sañudo-Wilhelmy *et al*. 2012
Riboflavin	0.2–5*	Sañudo-Wilhelmy *et al*. 2012
Thiamin	25–350*	Sañudo-Wilhelmy *et al*. 2012
Tryptophan	n.d.-1000^§^	Mopper and Lindroth 1982
Alanine	2000–70 000^§^	Mopper and Lindroth 1982; Lu *et al*. 2014
Methionine	n.d.-500^§^	Mopper and Lindroth 1982
Glycine	2000–100 000^§^	Mopper and Lindroth 1982; Lu *et al*. 2014
Glutamic acid	1000–20 000^§^	Mopper and Lindroth 1982; Lu *et al*. 2014
Glutamine	700–70 000^§^	Mopper and Lindroth 1982; Lu *et al*. 2014
Aspartic acid	2000–25 000^§^	Mopper and Lindroth 1982; Lu *et al*. 2014
Threonine	2000–14 000^§^	Mopper and Lindroth 1982
Asparagine	300–10 000^§^	Mopper and Lindroth 1982; Lu *et al*. 2014
Arginine	500–3000^§^	Mopper and Lindroth 1982
Serine	7000–47 000^§^	Mopper and Lindroth 1982
Valine	700–6000^§^	Mopper and Lindroth 1982
Histidine	1000–10 000^§^	Mopper and Lindroth 1982; Lu *et al*. 2014
Leucine	400–5000^§^	Mopper and Lindroth 1982
Lysine	1000–22 000^§^	Mopper and Lindroth 1982; Lu *et al*. 2014
Tyrosine	300–6000^§^	Mopper and Lindroth 1982
Vitamin B_12_	.1–8^¶^	Koch *et al*. 2011
Inositol	Data not available	
Adenine	Data not available	
Spermidine	1000–40 000^∞^	Nishibori *et al*. 2001; Lu *et al*. 2014
Uracil	Data not available	
Niacin	Data not available	

* Coastal California (1–800 m)

§ Baltic Sea (1–170 m); Coastal Georgia (2–17 m), note that these are dissolved free amino acids, amino acid availability is greater through protein degradation

• North Pacific Ocean (5–150 m)

¶ Gulf of Alaska (50–5500 m)

∞ Seto Inland Sea of Japan (surface, during phytoplankton blooms); Coastal Georgia (2–17 m)

In addition to the direct measurement of these compounds, molecular approaches have provided insight into the active transport, acquisition and need for these compounds in aquatic environments. Meta-omic approaches, such as metatranscriptomics and metaproteomics, have identified suites of genes/proteins that are likely being actively used for the sensing and transport of amino acids and vitamins (e.g. ABC transporters, TonB-dependent transporters, etc.) (Williams *et al*. 2012; Alexander *et al*. 2015; Bergauer *et al*. 2017). In addition, as we increase our understanding of biosynthetic pathways, metagenomic data can be leveraged to look for gaps in these pathways as was done to identify vitamin B_1_ auxotrophs in aquatic environments (Paerl *et al*. 2018). Moreover, advances in metabolomics make it a potentially powerful tool for the characterization of molecules in the environment (Kujawinski 2011; Moran *et al*. 2016). This approach facilitates characterization of novel auxophores in the environment. For example, untargeted metabolomics can detect the precursors to important biomolecules such as alpha-ribazole. Alpha-ribozole was detected in a culture of a marine bacterium (Johnson, Kido Soule and Kujawinski 2016) but currently only a non-marine bacterium is known to be auxotrophic for it (Gray and Escalante-Semerena 2010). Incorporating these tools with techniques using isotopically labeled molecules that could be traced through a community would help to further clarify the nature of these interactions (e.g. Ho *et al*. 2016; Mayali and Weber 2018). The continued use of multi ‘omic approaches will enhance our understanding of auxotrophy in aquatic environments as well as identify new auxophores for quantitative assessment and rate measurements.

**Table 3.** Experimental approaches to address open questions surrounding auxotrophy-based interactions (see section VIII for the formulation of the questions).

	Experimental approaches	Questions addressed
Sequencing	Metagenomic studies (e.g. MAGs)	1,2
	Single cell sequencing	1,5
	Genomic analyses of cultured isolates	2,4
Modeling	Population modeling	3,5
	Genome-scale metabolic modeling	4
	Incorporating auxotrophy into trait based model	3,5,6
Culturing	Synthetic consortia	1,2,3,4,5,6
	Chemostats, turbidostats	2,3,5,6
	Monoculture	4
	Stable-isotope tracing	1,4,6

## CASE STUDY

**Figure 3.**
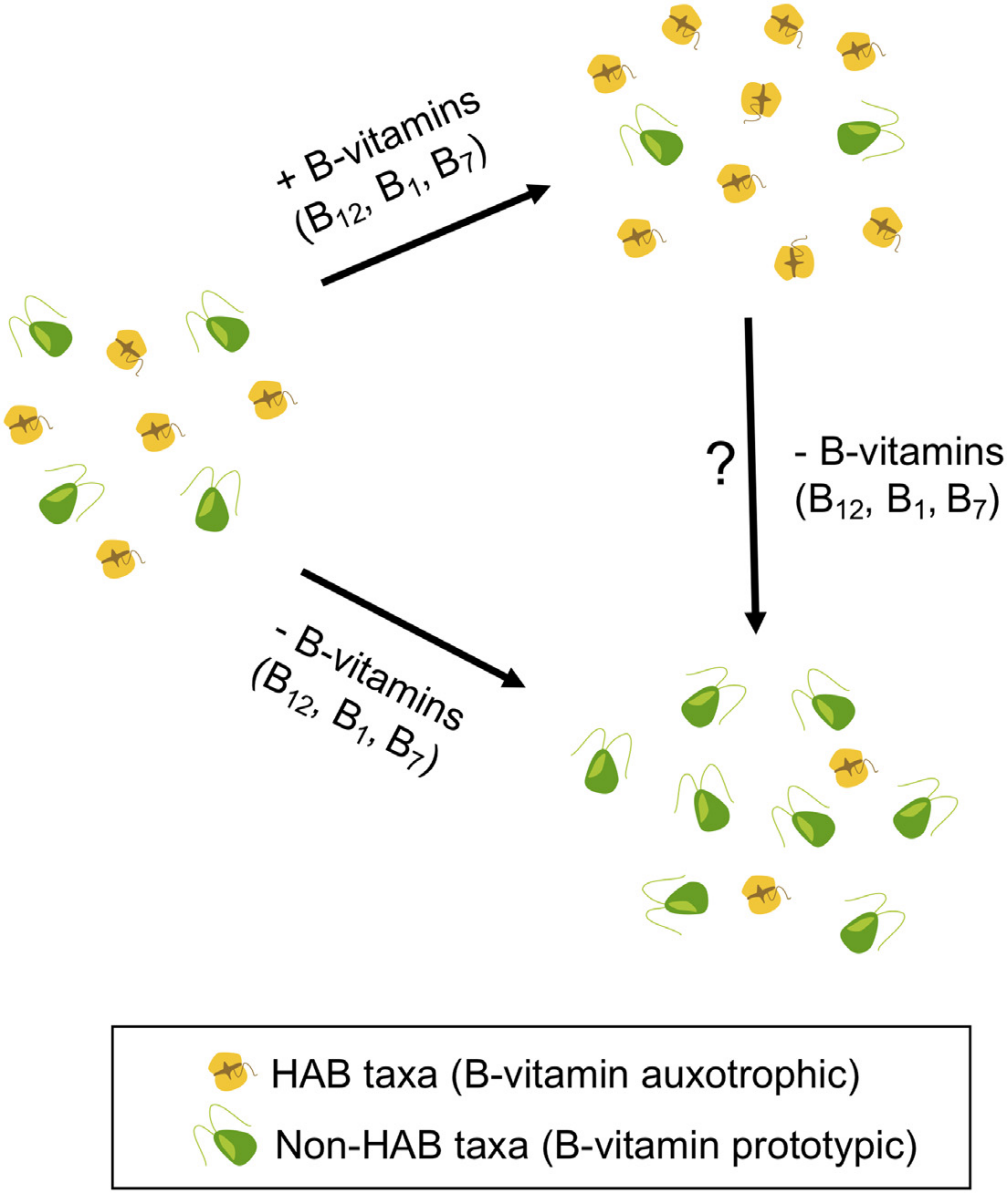
Case Study. B-vitamins shifting community stability via harmful algal blooms (HAB). An algal community containing taxa both with and without auxotrophy for B-vitamins can shift in composition depending on substrate availability. With the addition of B-vitamins, HAB taxa which are auxotrophic for B-vitamins can increase in population size while non-HAB taxa decline. When B-vitamin availability is reduced, the relative proportion of auxotrophic HAB taxa can decrease. Researchers are studying the effect of removing excess B-vitamins from aquatic environments during a harmful algal bloom to determine if HAB taxa will decrease.

While we have little direct evidence for how auxotrophic interactions might operate at the ecosystem level to stabilize aquatic communities, cases in nature may demonstrate connections between auxotrophy and community stability that could serve as a focal point for further inquiries. A prime example of this is the robustness of harmful algal blooms (HABs) which establish when toxic species (often monospecific, Anderson, Cembella and Hallegraeff 2012) become the dominant community taxa either as part of a natural cycle or resulting from anthropogenic influences. The prevalence of auxotrophy among some HAB taxa has been identified as one potential factor influencing the development and robustness of HABs (Tang, Koch and Gobler 2010). Specifically, if environmental conditions in non-HAB communities shift towards those that are favorable for auxotrophic organisms (*i.e*. the lacking substrate is supplied), then the previously non-HAB community can be replaced by HAB taxa (Fig. 3). Although we lack full information on the prevalence of auxotrophic taxa before this shift, HAB development may mark a transition from a community with relatively low auxotrophic abundance to one dominated by auxotrophs.

B-vitamin auxotrophy is common among HAB eukaryotes. Eukaryotes can acquire these essential vitamins through uptake of products which are bacterially synthesized or recycled from lysed phytoplankton (Croft *et al*. 2005; reviewed by Bertrand and Allen 2012). These sources of B-vitamins suggest that auxotrophic organisms do not rely on a single prototroph, thereby facilitating weaker interactions, which is expected to increase stability (Fig. 2). Tang, Koch and Gobler (2010) evaluated 45 species of dinoflagellate taxa (primarily HAB-forming species) for B-vitamin auxotrophy and found that over 90% of the taxa possessed an obligatory vitamin B_12_ (cobalamin) requirement and many also depended on external sources of B_1_ (thiamin) (49%) and B_7_ (biotin) (38%). While this nutrient requirement may be unremarkable in itself, the occurrence of B-vitamin auxotrophy in HAB taxa exceeds the 52% occurrence that is reported for all phytoplankton (Croft *et al*. 2005). Outcomes of vitamin depletion and addition experiments (Croft, Warren and Smith 2006; Sañudo-Wilhelmy *et al*. 2006; Bertrand *et al*. 2012) as well as temporal observations (Menzel and Spaeth 1962; Gobler *et al*. 2007; Vishniac and Riley 2011) support the idea of covariation between the supply of B-vitamins and shifts in population abundance of auxotrophic taxa, suggesting that B-vitamin availability may in part control the prevalence and robustness of blooms (Fig. 3). However, B_12_ availability has also been shown to affect phytoplankton that are not auxotrophic for B_12_: Swift and Guilford (1978) noted that when B_12_ was present some diatoms which dominate spring phytoplankton blooms grew faster and had short lag periods. Therefore even phytoplankton which can produce B_12_ may still benefit from importing it, but reserving the genetic ability to create B_12_ could expand their range of tolerable environments compared to B_12_ auxotrophs.

Once the community is dominated by HAB species, it can be difficult to shift away from that state (e.g. Sunda, Graneli and Gobler 2006; Reavie *et al*. 2017). This environmental challenge provides a unique opportunity to explore the role of auxotrophic HAB species in bloom robustness. Some of these cycles reflect instability through boom-bust dynamics and suggest a lack of negative feedbacks on the auxotroph or the producer (Allesina and Pascual 2008; Coyte, Schluter and Foster 2015). Disturbances, such as a substantial increase in nutrients, may also result in the community transitioning to an alternative stable state in which the community is dominated by HAB-forming algae and prone to recurrent algal blooms, for example microcystin-producing cyanobacteria in nutrient-impacted lakes (Downing, Watson and McCauley 2001). Though auxotrophy likely does not force HAB robustness, aquatic environments can be difficult to shift away from HAB-forming species (Anderson 2009) possibly due to a lack of strong negative feedbacks. Further, HAB organisms may perpetuate HAB events by exerting negative pressure on competitors (e.g. toxin-derived growth inhibition of other phytoplankton, unpalatability to zooplankton grazers (Turner and Tester 1989; Turner and Tester 1997; Turner *et al*. 1998; Sunda, Graneli and Gobler 2006), or recycling or regeneration of nutrients (Heisler *et al*. 2008), and this could lead to a more stable and resilient community.

Thus, while there may be a relationship between auxotrophy and stability within HAB-forming communities, comparing the nature of interactions between pre-HAB and HAB communities may require testing specific scenarios. Tang, Koch and Gobler's (2010) survey of wide-spread B-vitamin auxotrophy among HAB dinoflagellates suggests the potential for auxotrophic organisms to comprise a stable community through multiple interactions, though it is difficult to distinguish between auxotrophy and the effect of toxins that limit growth of other phytoplankton or grazing (Turner and Tester 1989; Turner and Tester 1997; Turner *et al*. 1998; Sunda, Graneli and Gobler 2006). The HAB-vitamin relationship is also likely more complex and during blooms may include nutrient co-limitation with, for example, N or Fe (Gobler *et al*. 2007; Koch *et al*. 2011; Bertrand *et al*. 2012). Thus, there is evidence to support the idea of a trend between auxotrophy and stability that occurs in HAB communities, yet greater genomic and experimental information for these scenarios is likely required to elucidate the important parameters for the relationship, the type and strength of the interaction and the determining factors of stability.

## FUTURE DIRECTIONS

With this discussion, we sought to outline the links between a type of organism-organism interaction, auxotrophy and its ability to stabilize or destabilize an aquatic microbial community in the face of environmental changes. We posit that the influence of auxotrophy on community stability hinges on whether auxotrophy causes stronger or weaker species interactions in the overall community. We predict that enhanced stability will be found in communities with (i) complex and redundant auxotrophic interactions that have more numerous higher-order cooperative interactions among producer and receiver organisms or (ii) a net negative feedback on the producer that would as a result keep the growth of the auxotroph in check. Identifying the extent to which these predictions occur across space and time may provide explanatory power for evaluating ecosystem stability and community structure in cases where current frameworks, such as generalist-specialist divisions and functional redundancy, alone are incomplete.

Broadly, research on microbial community cross-feeding and auxotrophy is currently a thriving and essential field with important implications in many ecosystems. As demonstrated in this article (Fig. 2), there remain many open questions about this relationship and how interaction strength, directionality, connectivity, and complexity can influence ecosystem biomass and diversity, as well as its stability. We suggest that opportunities exist to continue examining the strength of various auxotroph-prototroph relationships, the degree of connectivity between the auxotrophs and other community members, the degree to which auxotrophy enhances positive feedback loops, the rate at which an essential molecule can be transported into the cell and the extent to which a specific auxotrophy confers metabolic advantage.

Below we specify some of the open questions related to these opportunities for use as a guide for efforts to understand the role of auxotrophy in community structure and ecosystem stability. We also include suggestions for practical approaches corresponding with the questions below (Table 3).

What types of interactions (e.g. mutualism, commensalism, parasitism) most commonly result from auxotrophy? What biotic or abiotic conditions foster each type of interaction?
What are the relationships between auxotrophy, genome streamlining, and functional redundancy in a population and in a community?
What is the quantitative threshold between a weak and strong auxotrophic interaction and when does crossing that threshold lead to a shift in stability?
Are certain biochemical molecules more likely to be auxophores given that they confer a greater advantage based on cost of synthesis or are easily transported into the cell?
To what extent does auxotrophy create positive or negative feedback loops? Over evolutionary time, how likely are these loops to shift directions or disintegrate?
How are the rates of microbial processes affected by changes to community stability as a consequence of auxotrophic interactions?

New developments in tools for researching microbial communities provide unique opportunities to test and develop theories for the existence and creation of relationships between auxotrophy and community structure or ecosystem stability. We believe that, as a research community, we can leverage these new tools to generate insights into the significance of auxotrophy in community dynamics (Table 3). Ultimately, these insights will help develop a robust ecological theoretical framework that accounts for the dynamic metabolic uniqueness and interconnectedness of microorganisms (both providers and recipients of essential molecules). Addressing the above questions will propel us towards that goal by increasing our understanding of auxotrophy as a mediator of community stability and composition. Moreover, deeper study and consideration of the role of auxotrophy could transform our conception of what characterizes stable and resilient microbial communities. In the light of pressing environmental change, understanding how auxotrophy shapes communities by tuning and defining these transient yet obligatory microbial interactions is paramount.
